# Supplementary material for: Nitrates as a Potential N Supply for Microbial Ecosystems in a Hyperarid Mars Analog System
Source: Life (Basel). 2019 Oct 19;9(4):79. doi: 10.3390/life9040079 (PMC6958444; doi:10.3390/life9040079)
Supplement: Supplementary file 1 [file life-09-00079-s001.pdf]

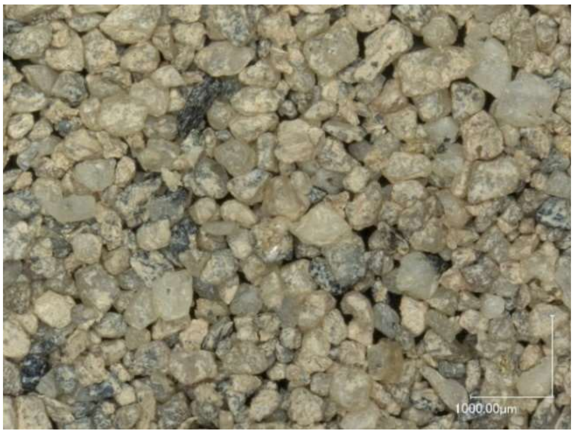

(**a**)

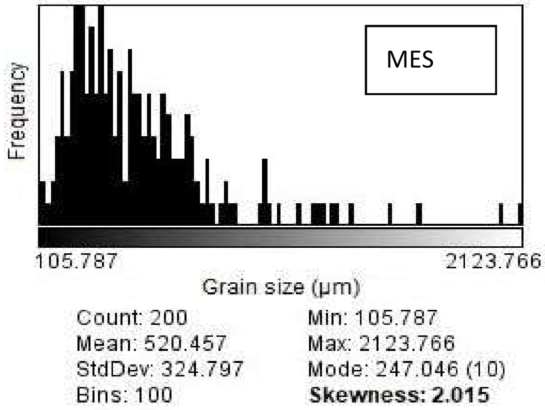

(**b**)

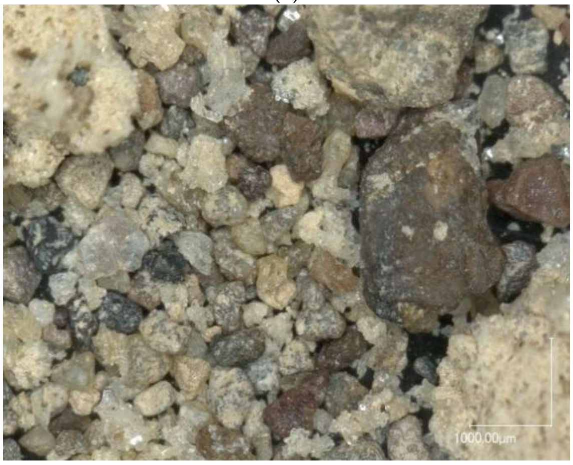

(**c**)

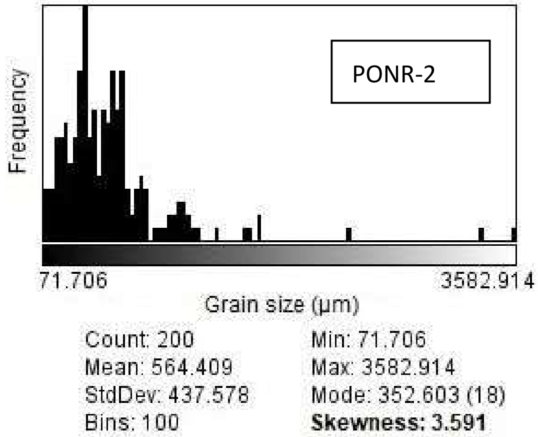

(**d**)

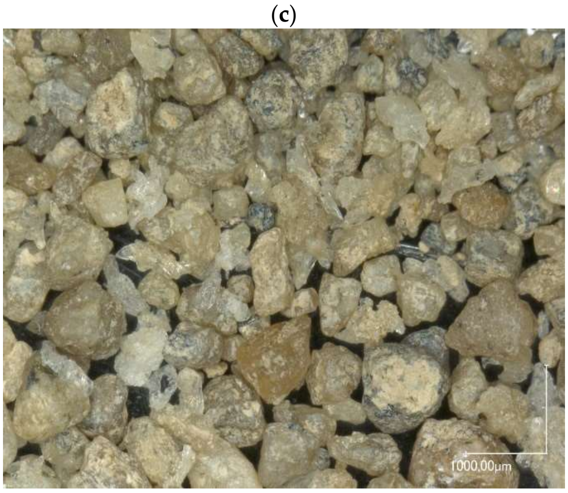

(**e**)

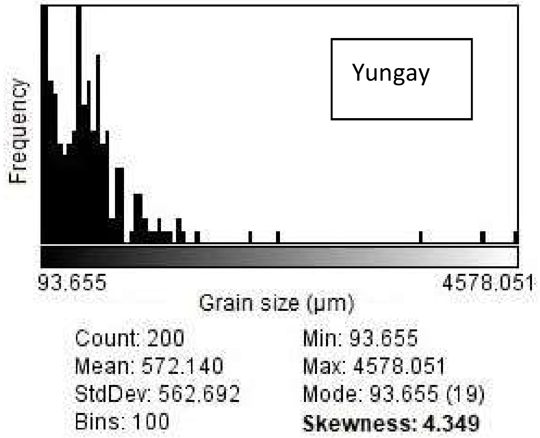

(**f**)

Figure S1. *Cont.*

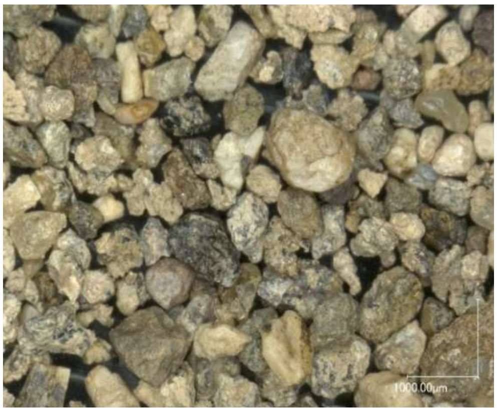

(**g**)

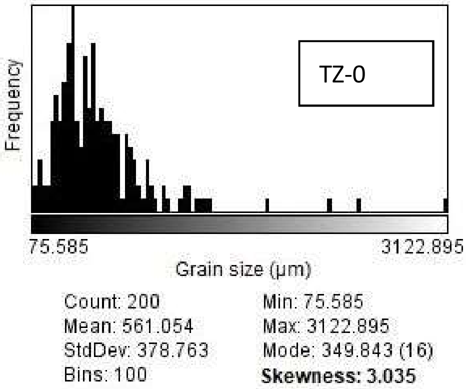

(**h**)

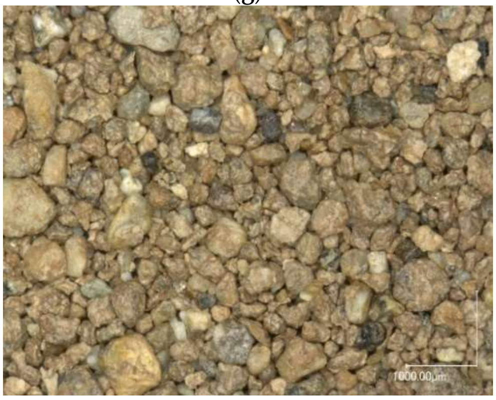

(**i**)

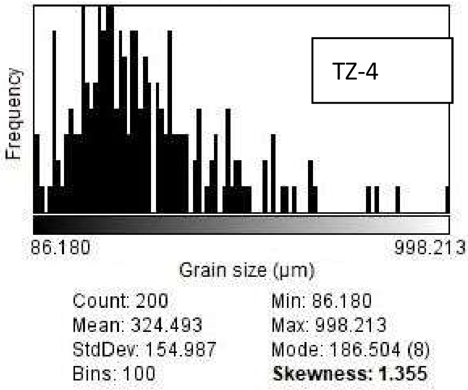

(**j**)

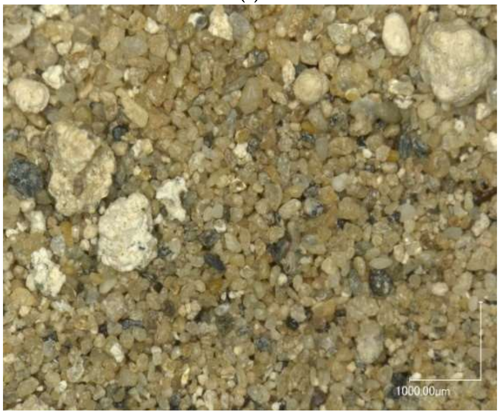

(**k**)

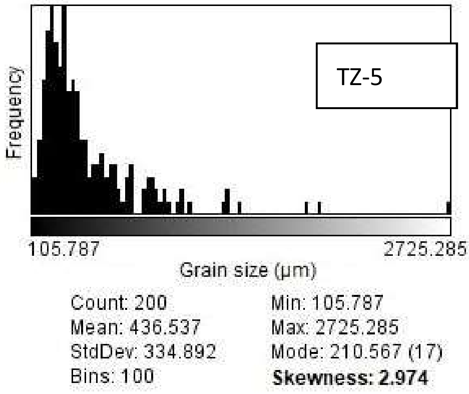

(**l**)

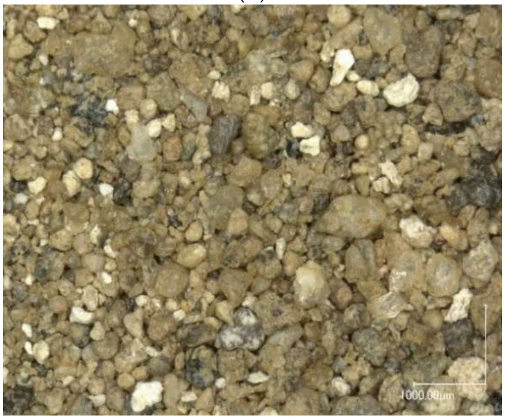

(**m**)

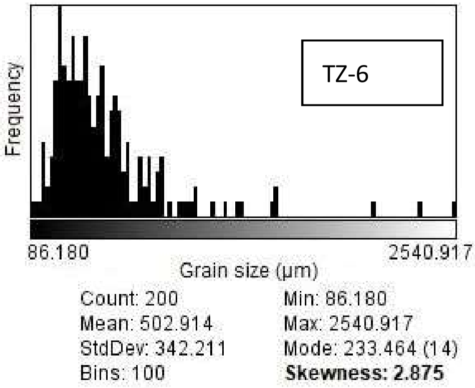

(**n**)

Figure S1. Microscopic visualization and grain size distribution of (**a**,**b**) MES, (**c**,**d**) PONR-2, (**e**,**f**) Yungay, (**g**,**h**) TZ-0, (**i**,**j**) TZ-4, (**k**,**l**) TZ-5, and (**m**,**n**) TZ-6.

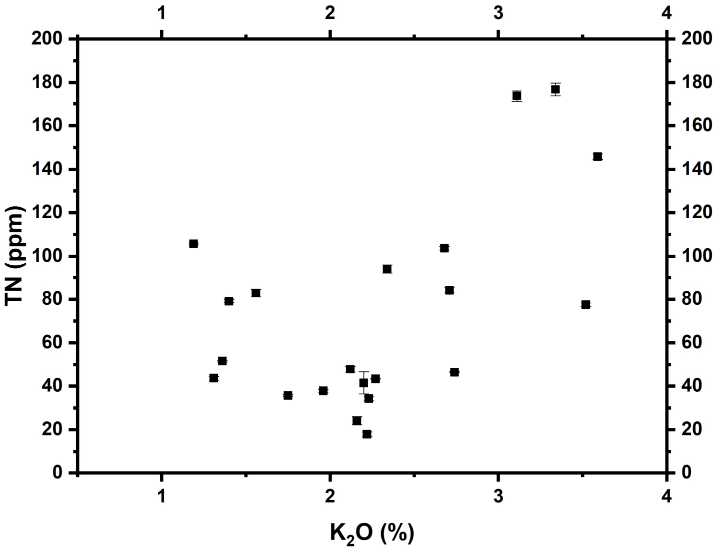

Figure S2. Scatter plot of TN and potassium. Errors on K_2O measurements were smaller than the symbols.

Table S1. Detailed major element components of sampling pits. The standard deviation of each measured major sedimentary elements is between 0.01% and 0.05% based on precision analyses of XRF standards (LOI, loss on ignition).

Pit Name	SiO_2 %	TiO_2 %	Al_2O_3 %	Fe_2O_3 %	MnO %	MgO %	CaO %	Na_2O %	K_2O %	P_2O_5 %	LOI %	Cl %
M1	69.05	0.39	14.84	2.92	0.05	1.27	3.31	4.19	2.22	0.13	1.43	0.011
M2	62.48	0.68	14.95	5.29	0.09	2.22	4.56	3.55	2.12	0.16	3.49	0.008
M3	67.94	0.36	14.81	2.81	0.05	1.34	3.79	3.94	2.16	0.12	2.38	0.010
P1	64.05	0.74	15.23	4.44	0.08	1.27	3.80	2.93	2.74	0.26	3.86	0.009
P2	51.48	0.60	13.37	4.26	0.07	1.65	8.57	2.41	2.34	0.20	13.11	0.005
P3	33.78	0.52	8.80	3.29	0.06	1.03	16.91	1.69	1.31	0.11	17.36	0.006
Y1	47.01	0.67	12.14	3.68	0.06	1.21	11.83	3.31	2.27	0.08	10.41	0.008
Y2	47.88	0.48	12.08	2.88	0.06	1.09	11.24	2.90	2.23	0.09	14.65	0.007
Y3	52.45	0.78	12.93	4.23	0.07	0.99	9.57	3.48	2.20	0.11	7.96	0.009
T01	53.32	0.98	13.62	6.63	0.24	3.43	6.72	3.29	1.96	0.23	9.34	0.007
T02	49.66	0.79	12.02	8.17	0.23	4.81	7.99	3.05	1.36	0.18	11.52	0.007
T03	53.48	0.78	14.20	6.75	0.23	3.71	6.29	3.79	1.75	0.17	8.59	0.008
T41	57.88	0.65	15.40	5.39	0.13	2.78	3.54	2.54	2.71	0.38	8.38	0.005
T42	53.33	0.58	15.02	5.18	0.14	3.21	5.28	2.09	2.68	0.44	11.80	0.006
T43	54.01	0.71	14.76	6.60	0.15	3.30	4.05	3.01	3.52	0.35	9.20	0.005
T51	55.99	0.55	12.47	5.49	0.08	1.92	10.92	3.46	1.56	0.34	6.93	0.013
T52	55.79	0.63	12.57	5.84	0.09	2.06	9.67	3.96	1.40	0.36	7.37	0.024
T53	44.37	0.49	9.92	5.21	0.07	2.70	17.26	3.03	1.19	0.37	14.75	0.018
T61	57.35	0.86	13.56	9.47	0.17	2.86	3.34	3.12	3.59	0.22	5.17	0.010
T62	50.10	0.71	11.50	8.02	0.13	2.52	9.38	3.66	3.34	0.14	10.18	0.014
T63	52.25	0.76	12.09	7.70	0.13	2.62	8.48	2.70	3.11	0.20	9.65	0.017
